# RETRACTION: The Use of Bacteria in Cancer Treatment: A Review from the Perspective of Cellular Microbiology

**DOI:** 10.1155/emmi/9853270

**Published:** 2026-10-06

**Authors:** Emergency Medicine International

RETRACTION: H. Mills, R. Acquah, N. Tang, et al., “The Use of Bacteria in Cancer Treatment: A Review from the Perspective of Cellular Microbiology,” *Emergency Medicine International* 2022, no. 1 (2022): 8127137, https://doi.org/10.1155/2022/8127137.

The above article, published online on 08 August 2022 in Wiley Online Library (https://wileyonlinelibrary.com), has been retracted by agreement between the journal Editor‐in‐Chief, Roberto Cirocchi and John Wiley & Sons Ltd. The retraction has been agreed due to concerns raised about the existence of the authors, which calls into question the integrity of the research presented.

Several attempts by the journal to contact the authors have not resulted in a reply.

